# Enabling conditions for conservation on Indigenous and community lands

**DOI:** 10.1111/cobi.70055

**Published:** 2025-06-19

**Authors:** Stephanie Brittain, Andrea Alatorre, Leigh‐Anne Bullough, Helen Newing

**Affiliations:** ^1^ Department of Biology University of Oxford Oxford UK; ^2^ Forest Peoples Programme Moreton‐in‐Marsh UK

**Keywords:** community‐based conservation, enabling conditions, Indigenous Peoples and local communities, post‐2020 framework, rights‐based approaches, comunidades locales y pueblos indígenas, condiciones habilitantes, conservación comunitaria, estrategia basada en derechos, marco post‐2020

## Abstract

Despite increasing evidence and general acceptance in global environmental policy of the significant role of Indigenous Peoples and local communities (IP&LC) in biodiversity conservation and climate change mitigation, an implementation gap remains between global policy and how conservation plays out on the ground. One reason for this discrepancy may be the lack of a coherent evidence base on how best to support the contributions of IP&LC to conservation. Enabling conditions are often discussed in conservation policy, but the diverse factors that may enable or disable Indigenous and community conservation are frequently not considered in empirical studies of conservation outcomes. We explored the enabling conditions and ecological outcomes of conservation that are measured or reported in the literature on forested lands held by IP&LC and identified gaps and biases in the current knowledge base. We searched 3 bibliographic databases and screened the results for relevance against predefined inclusion criteria, reviewing 182 articles. Articles examined the effects of 20 enabling conditions on 11 ecological outcomes. The more frequently explored links were between the enabling conditions—governance and law and policy—and the outcomes—forest cover and forest quality. Key knowledge gaps were the impacts of enabling conditions on species‐level outcomes and certain ecosystem services, such as soil and water quality and carbon sequestration. Priorities for future reviews include in‐depth examinations of the linkages we identified and the quality of evidence that exists. Understanding how IP&LC can best be supported is a critical step in promoting rights‐based approaches, as set out in the post‐2020 Global Biodiversity Framework.

## Abstract (es)

Condiciones habilitantes para la conservación en tierras comunitarias e indígenas

**Resumen:**

A pesar de la creciente evidencia y aceptación general en la política ambiental mundial del importante papel de los pueblos indígenas y las comunidades locales en la conservación de la biodiversidad y la mitigación del cambio climático, todavía existe una brecha entre la política mundial y la forma en que la conservación se lleva a cabo en la práctica. Una de las razones de esta discrepancia puede ser la falta de una base empírica coherente sobre la mejor manera de apoyar las contribuciones de los pueblos indígenas y las comunidades locales a la conservación. En las políticas de conservación se suele hablar de condiciones favorables, pero los diversos factores que pueden permitir o impedir la conservación indígena y comunitaria no suelen considerarse en los estudios empíricos sobre los resultados de la conservación. Analizamos las condiciones favorables y los resultados ecológicos de la conservación que se miden o de los que se informa en la bibliografía sobre las tierras forestales de los pueblos indígenas y las comunidades locales, e identificamos los vacíos y los sesgos en la base de conocimientos actual. Buscamos en tres bases de datos bibliográficos y analizamos los resultados en función de criterios de inclusión predefinidos, en la revisión de 182 artículos. Los artículos analizaban los efectos de 20 condiciones favorables sobre 11 resultados ecológicos. Los vínculos más frecuentemente explorados fueron entre las condiciones favorables de gobernanza, legislación y política, y los resultados de cubierta forestal y calidad de los bosques. Las principales lagunas de conocimiento fueron los efectos de las condiciones favorables sobre los resultados a nivel de especie y determinados servicios ecosistémicos, como la calidad del suelo y del agua y la captura de carbono. Las prioridades para futuras revisiones incluyen exámenes a profundidad de los vínculos que identificamos y la calidad de las pruebas existentes. Comprender cuál es la mejor manera de apoyar a los pueblos indígenas y las comunidades locales es un paso fundamental para promover enfoques basados en los derechos, como se establece en el Marco Mundial para la Diversidad Biológica posterior a 2020.

## INTRODUCTION

The importance of forests for the health, well‐being, and livelihoods of Indigenous Peoples and local communities (IP&LC) is undisputed (CBD, 2022; IPBES, 2019). Globally, an estimated 1.6 billion people live in and around forests, two thirds of whom live in tropical and lower‐ and middle‐income countries (Newton et al., 2020). Forests are crucial for biodiversity conservation and climate mitigation, covering almost one third of the global land area and hosting more than three quarters of the world's terrestrial life (Hill et al., 2019; Walcott et al., 2020). Forests comprise a key global natural resource and contribute to several of the UN Sustainable Development Goals, including reduced poverty, zero hunger, and improved health and well‐being (Cheng et al., 2019; Miller et al., 2020; Timko et al., 2018). They also provide essential ecosystem services including clean water, food, and culturally important sites that can in turn promote improved human health, empowerment, cultural integrity, and well‐being (Cheng et al., 2019; Raymond et al., 2009). There is growing evidence of the global conservation significance of lands held by IP&LC (Dawson et al., 2021; Fa et al., 2020; Garnett et al., 2018). At least one quarter of the total global land area held by IP&LC overlaps significantly with biodiversity‐rich areas, with biodiversity and ecosystem services on their lands declining less rapidly than elsewhere (IPBES, 2019). IP&LC play a crucial role in forest conservation; an estimated 36% of intact forest landscapes overlap with Indigenous Peoples’ lands (Fa et al., 2020).

However, despite the general acceptance of the significant role of IP&LC in biodiversity conservation and climate change mitigation, including in global environmental policy, and despite increasing evidence of the link between Indigenous and community control and positive ecological outcomes (Dawson, Coolsaet, Bhardwaj, Booker, et al., 2024; Dawson, Coolsaet, Bhardwaj, Brown, et al., 2024), an implementation gap remains between policy, conservation finance, and how conservation plays out on the ground (Corson et al., 2020). Conservation finance directed at supporting Indigenous and local community contributions to conservation efforts is still dwarfed by the contributions made to government and private projects, and conservation on the ground continues to be dominated by protected area expansion, which still often involves the displacement and exclusion of IP&LC (Hatcher et al., 2021; Smith et al., 2019; Tauli‐Corpuz et al., 2020).

One reason for this discrepancy may be the lack of a strong evidence base demonstrating how best to support contributions by IP&LC to conservation. Although efforts are being made to synthesize evidence of the impacts that IP&LC have on conservation outcomes (Busch & Ferretti‐Gallon, 2023), studies that explore the enabling conditions required to support contributions to conservation are lacking.

Following Huber‐Stearns et al. (2017), we define *enabling conditions* as the environmental, economic, governance, and social–cultural factors that increase the likelihood of successful conservation outcomes, such as retained or increased forest cover, floristic diversity, or species richness. Multiple fields have addressed the concept of enabling conditions including economics (Coase, 1960; Jack et al., 2008), political science (Agrawal, 2001; Ostrom, 2009; Sabatier, 1986), and ecology (Rands et al., 2010). The term has gained prominence in environmental policy and conservation discourse as a way to articulate the contextual factors that support or hinder effective and sustainable natural resource management by communities, Indigenous Peoples, or stakeholders.

Contextual enabling conditions encompass a spectrum of elements, including those related to legal frameworks, governance structures, policy support, access to resources, financial mechanisms, and social networks. For instance, secure land tenure rights can be considered an enabling condition because they empower communities to defend their lands against encroachment and invest in conservation efforts without the fear of land loss. Similarly, supportive policies that recognize traditional knowledge can create an enabling environment for culturally informed conservation practices. For example, Hill et al. (2012) highlight key conditions that are crucial for ensuring the effectiveness and sustainability of IP&LC contributions to conservation efforts. For successful conservation, these include the recognition of Indigenous ecological knowledge (IEK), a collaborative governance approach, respect for Indigenous cultural values, capacity building, and adaptive management that accommodates traditional practices. Tengö et al. (2014) further underscore the importance of knowledge sharing as an enabling condition for effective IP&LC contributions to conservation. They advocate for a multiple‐evidence‐based approach, emphasizing the integration of diverse knowledge systems. This approach values Indigenous and local knowledge alongside scientific knowledge, creating a more holistic understanding of ecosystems. Conceptualizing enabling conditions allows policymakers and researchers to understand and address the complexities of community‐based conservation by emphasizing the importance of contextual factors in achieving successful outcomes.

Other authors include community characteristics under the framing of enabling conditions that make it more likely that collective actions will result in positive environmental outcomes. For example, Brooks et al. (2012) found that community characteristics including supportive cultural beliefs and institutions are important for achieving positive conservation outcomes and that collaborative and well‐designed projects that consider these characteristics can prevail over disadvantageous national contexts. Many in conservation also believe that when communities manage their own resources, their efforts are more effective than top‐down approaches (Sandbrook et al., 2019).

Although enabling conditions have long been discussed in policy fora, and the rights of IP&LC are recognized in policy at the international level, these conditions are not always adequately supported in the way that conservation research and practice takes place on the ground (Brooks et al., 2012). As a result, there is only a limited, fragmented evidence base from which to improve and support the design of conservation practice. We reviewed the literature that examines how the presence or absence of different enabling conditions supports positive ecological outcomes from Indigenous and local conservation efforts. We focused particularly on papers that provide evidence of the enabling conditions that influence the ability of IP&LC to conserve forested land effectively.

We devised a systematic map of the academic conservation literature to explore enabling conditions and ecological outcomes of conservation on forested lands held by IP&LC and identified gaps and biases in the current knowledge base that require further research. The broad scope of our study provides an opportunity to clarify and strengthen understanding of the contributions IP&LC make to conservation and how they can be supported in doing so. Ultimately, this understanding is vital for informing forest and conservation‐related policy, research, investment, and evidence‐based action.

## METHODS

### Systematic mapping

Systematic maps are overviews of the distribution and abundance of evidence in relation to multifaceted elements of a broad question of policy or management relevance and are used to collate and describe a body of literature across a subject of interest (James et al., 2016; Pullin et al., 2022). Similar to a scoping review, a systematic map intends to answer broad questions and build a catalogue of evidence to identify knowledge gaps and trends, specifically, what research has been conducted where, how, and in what form (Haddaway et al., 2016; Munn et al., 2018). We conducted a systematic map because the academic literature on the conditions for positive conservation outcomes on IP&C lands had not yet been synthesized and to identify specific questions for deeper review. We maintained a narrow focus on ecological outcomes (rather than including social outcomes as well) so that the review could directly inform contemporary debates about the ecological effectiveness of IP&LC stewardship.

We focused on the academic literature because we wanted to explore how enabling conditions are being conceptualized principally by academic researchers and the degree to which assumptions about enabling conditions are being evaluated through the collection of evidence consistently and comparably. However, publications other than peer‐reviewed articles (such as reports, theses, and books) may represent a greater degree of knowledge coproduction between academics and nonacademics, which is essential for moving beyond conceptualizations and toward addressing conservation challenges (Norström et al., 2020).

### Questions and objectives

Our overarching goal was to explore what enabling conditions researchers are discussing and to what extent they are gathering evidence to support the impact of enabling conditions on ecological outcomes. We developed a set of questions to help identify key concepts, sources, and knowledge gaps: What is the scope of the published research (e.g., how much research is available, in which journals, and what are the temporal and geographical publication trends); what are the enabling conditions and ecological outcomes that are most commonly described and linked in the literature; and what are the characteristics of the published literature (e.g., are there trends or gaps in the enabling conditions and types of conservation outcomes that are frequently discussed in the literature)? Answering these questions provides an overview of the existing knowledge landscape in IP&LC contributions to conservation. It helps identify research gaps, best practices, and areas where more attention is needed. This, in turn, can inform the development of more effective policies and strategies that bridge the implementation gap between global environmental policy and on‐the‐ground contributions to conservation by IP&LC.

### Searches

**FIGURE 1:**
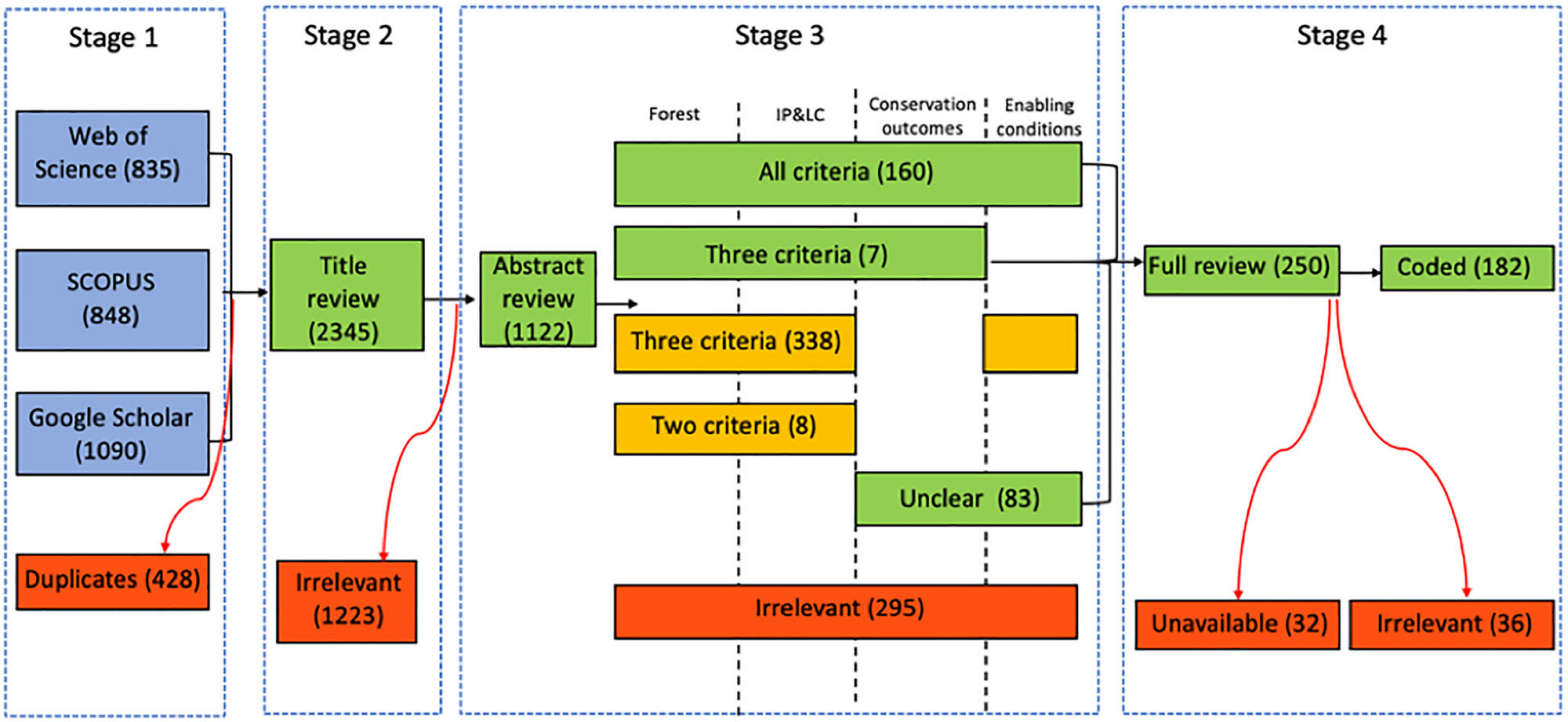
Summary of each stage of the review of the enabling conditions for conservation on Indigenous and community lands, including the number of articles at each stage (green, papers carried through to the next stage; red, papers removed from the review process).

Our search strategy consisted of 4 stages: identification, title screening, abstract screening, and inclusion (Figure 1). During the identification stage, we collated a draft list of English‐language terms that would enable us to answer the research questions, based on a preliminary scoping review of the literature. The search was restricted to English‐language publications because of limited resources, although this limits the generalizability of the findings (for further discussion of this issue, see Amano et al. [2021]). Once initial agreement on the search terms was reached by the authors and with input from our collaborators from the Forest Peoples Programme, we ran tests with different combinations of these terms, on 3 search engines: Web of Science, SCOPUS, and Google Scholar. Following the tests, we assessed the results and refined the search terms to ensure they were inclusive of key papers we were aware of but specific enough to produce a manageable dataset (see Table 1 for slight variation in use between databases).

**TABLE 1:** Search terms used in each of the 3 databases used in a review of the enabling conditions for conservation on Indigenous and community lands.

Database	Search term
Google Scholar	“enabling conditions” OR succe* OR fail* OR context OR preconditions AND “local community” OR IPLC OR indigenous OR “collective action” AND conservation OR “sustainable use” OR CBNRM OR “community forest” OR “social forestry” OR “community‐based forestry” AND forest*
SCOPUS	(TITLE‐ABS‐KEY (“enabling conditions” OR succe* OR fail* OR context OR preconditions) AND TITLE‐ABS‐KEY (“local community” OR iplc OR indigenous OR “collective action”) AND TITLE‐ABS‐KEY (“conservation” OR “sustainable use” OR cbnrm OR “community forest” OR “social forestry” OR “community‐based forestry”) AND TITLE‐ABS‐KEY (forest*))
Web of Science	TOPIC: (“enabling conditions” OR succe* or fail* OR context OR preconditions) AND TOPIC: (“local community” OR IPLC OR indigenous OR “collective action”) AND TOPIC: (“conservation” OR “sustainable use” OR CBNRM OR “community forest” OR “social forestry” OR “community‐based forestry”) AND TOPIC: (forest*)

Literature searches were carried out from March 2020 to May 2020 and updated from September to November 2021 for new articles released after May 2020. We extracted all the results from Web of Science and SCOPUS and from the first 99 pages in Google Scholar with Mendeley's Web Importer.

### Article screening

During the screening stage, duplicates were first removed from the data set. Articles not written in English were excluded. The titles and abstracts of the articles were screened based on 4 predefined selection criteria. Publications were selected that referred to IP&LC (Criterion 1) and that were based on a case study or research relating to forested lands (Criterion 2). Articles also had to allude to a discussion of positive or negative conservation outcomes on IP&LC land (Criterion 3) or indicate potential enabling conditions for conservation (Criterion 4). If Criterion 1 and Criteria 2 or 3 were met, the abstract of the paper was reviewed. If there was uncertainty, the abstract was reviewed.

The first 300 titles were screened by A.A., L.‐A.B., and S.B. to evaluate consistency. Cohen's kappa coefficient for reliability demonstrated strong agreement between the reviewers (0.79), indicating that the criteria for accepting and rejecting articles based on titles was clear and consistent. Once the title screening was complete, we downloaded the abstracts or executive summaries of all the accepted titles for review against the 4 criteria. The first 100 abstracts were reviewed by A.A., L.‐A.B., and S.B., and 2 Kappa tests were conducted until strong agreement was achieved (0.72). Finally, we screened the full text of the articles.

### Thematic analyses

We prioritized articles that met all the selection criteria and uploaded the full articles into the qualitative analysis software NVivo 12 for coding (QSR International 2018). We entered the following case attributes for each article: type of article (e.g., case study, an NGO [nongovernmental organization] report, review); lead author institution (e.g., NGO, university, research institute, government organization); country (or countries) in which the research was carried out; and year of study, so that the conditions emerging from the literature could be compared spatially and temporally.

We then carried out a thematic analysis of enabling conditions and conservation outcomes with a grounded theory approach. Grounded theory involves constructing theories through the methodical gathering and analysis of data (Glaser & Strauss, 1967). Distinguished from traditional hypothesis‐driven research, a fundamental of the grounded theory approach is its emphasis on the generation of theories that are directly grounded in the data itself (Charmaz, 2006). This approach is particularly invaluable in areas with limited preexisting knowledge. Grounded theory's inductive nature, which focuses on deriving categories, themes, and patterns directly from data, equips researchers to develop a deeper, more intricate understanding of the subject under investigation (Bryant & Charmaz, 2007). The iterative process of grounded theory, continuing to develop and refine the themes at successive levels of detail until the body of evidence can be coherently presented (Snilstveit et al., 2012), allows the construction of hypotheses, conceptual frameworks, and theories through the collecting and analysis of data (Strauss & Corbin, 1994).

We scanned titles and abstracts and noted the different kinds of enabling conditions discussed and the different kinds of ecological outcomes reported. We then consolidated the resulting long lists of enabling conditions and ecological outcomes into a set of themes with a grounded theory approach. This involved a process of noting recurring themes, developing codes as markers for them, with conceptual definitions where necessary, and then lumping, splitting, or more precisely defining the different codes in an iterative process until they form a coherent conceptual set.

The themes that emerged from this process were used in the subsequent analyses as high‐level codes (nodes in NVivo) for different conditions and outcomes. We also noted the evidence provided and whether the outcome was positive or negative. The high‐level codes were then further refined, and a more detailed coding set was developed through analysis of the full text of the articles, again with a grounded theory approach.

Robustness and replicability in thematic analyses have nothing to do with what codes are developed, which is related partly to the scope and focus of the study, but rather with whether the codes are applied consistently. The Kappa tests indicated a high level of consistency in the initial stages of analysis and therefore confirmed robustness and replicability in the application of the 4 initial criteria.

Given the large volume of articles included and time and financial constraints, each article was initially coded by a single researcher, but articles where the coding was uncertain or unresolved were regularly brought to the full research team to ensure collective agreement. During the initial stages of writeup, some minor adjustments were made to the codes to address any remaining ambiguities or overlaps between the identified codes. These adjustments were then applied back to the data set.

The final codebook is in Appendix S1. We mapped the coverage of different enabling conditions and outcomes in matrices to identify key knowledge gaps that require further research. Given the broad scope of this study, we did not attempt to critically appraise individual studies.

## RESULTS

The systematic search resulted in 2345 articles for title screening, after accounting for duplicates between databases. Title and abstract screening further reduced the number of potential records. We retained the 160 articles that met all 4 selection criteria and 7 articles that met Criteria 1, 2, and 3 but did not mention enabling conditions explicitly. We also retained the 83 articles that were clearly linked to forests and IP&LC but required further reading to ascertain their relevance for conservation outcomes or enabling conditions. Of those 250 articles, 32 were unavailable online, and a further 36 were deemed irrelevant upon full review (e.g., they did not mention conservation outcomes or enabling conditions). Therefore, 182 articles were included in the final map (Figure 1).

**FIGURE 2:**
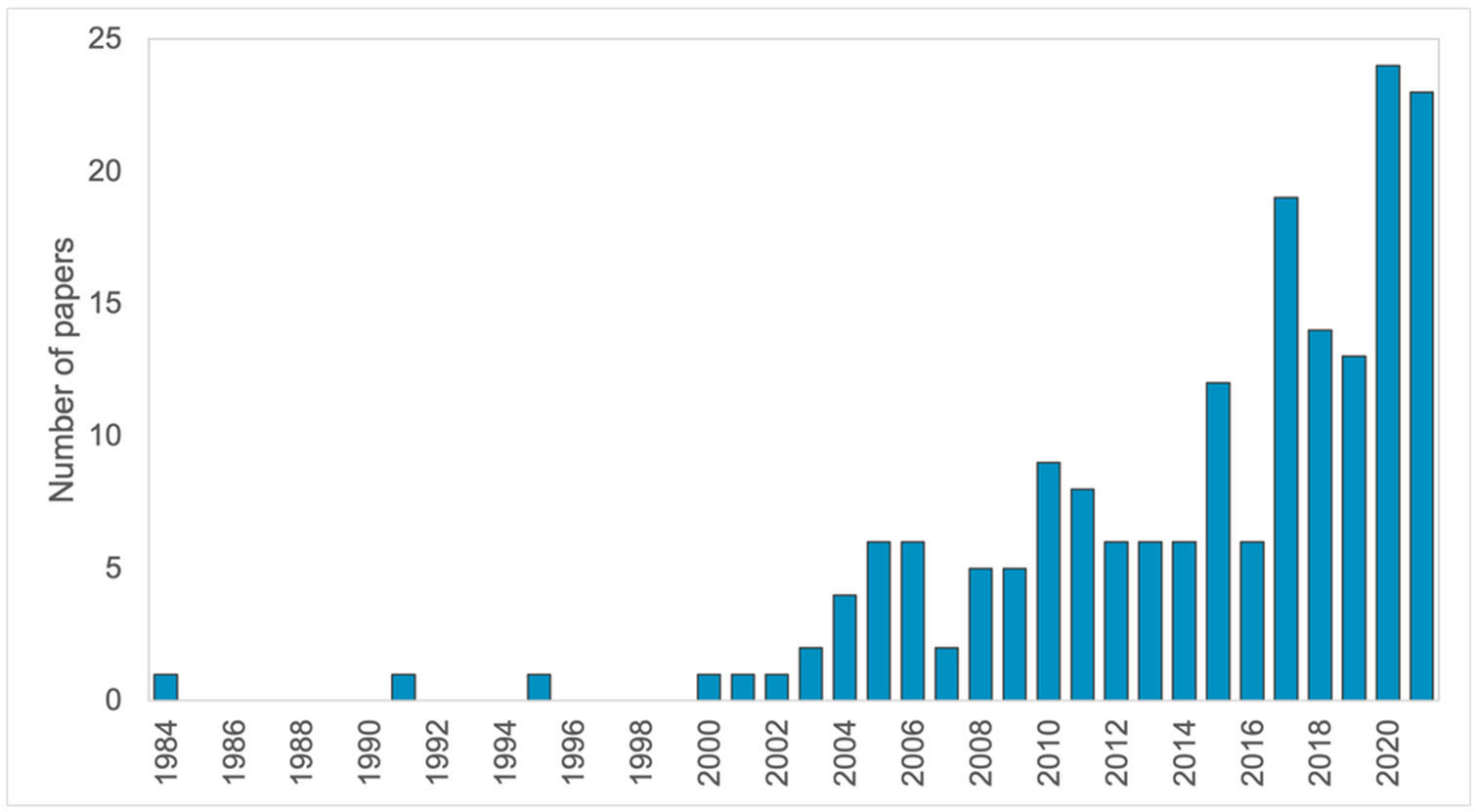
Number of publications included in the review of the enabling conditions for conservation on Indigenous and community lands by publication year.

A total of 84 different journal outlets were included in this systematic map, of which 19 published 3 or more of the articles included in this study (Appendix S2). The oldest paper was published in 1984. Few papers were published prior to 2000. The volume of articles published increased from the year 2002 (Figure 2), a trend that has been found in other systematic maps and reviews of the role of nature in human well‐being and the role of Indigenous Peoples in equitable conservation (Busch & Ferretti‐Gallon, 2023; Dawson et al., 2021; McKinnon et al., 2016).

Forty‐eight (26%) of the included papers used the term *enabling condition* or *enabling conditions*. The first mention of the term was in 2008. It became more consistently used from 2015 onward; 40 (83%) of the studies that mentioned enabling conditions were published from 2015 onward.

**FIGURE 3:**
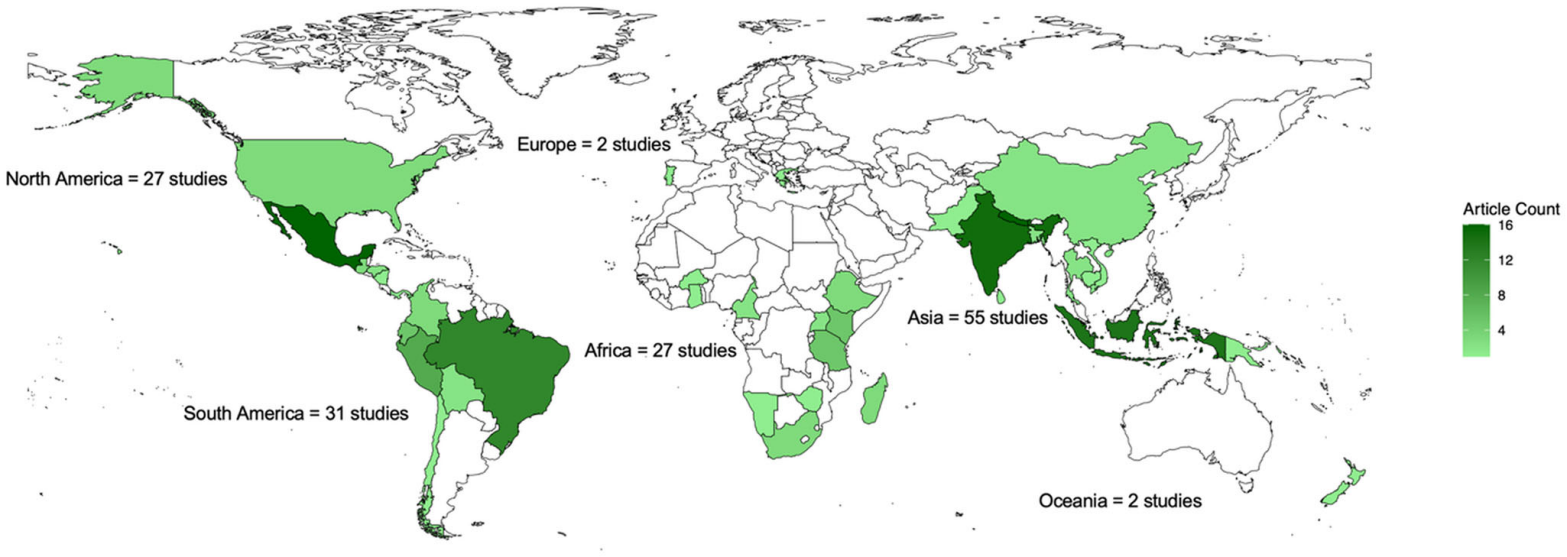
Geographic distribution of the study locations included in the review of the enabling conditions for conservation on Indigenous and community lands (includes 144 of the 182 articles identified). A further 24 studies were conducted in multiple countries or regions, and 7 had a global scope.

The articles we included in the analyses described studies in all continents except Antarctica, with the greatest number in Asia, followed by the Americas and Africa (Figure 3). Forty‐three countries were represented. The countries with the highest number of papers were Nepal, India, Mexico, Indonesia, Brazil, and Peru, which, combined, accounted for 45% of the papers (*n* = 81). We did not explore the reasons for the concentration of research effort in these countries further but presumed that it reflected a mixture of the prevalence of community‐based conservation and community forestry and a pervasive asymmetry in knowledge generation that is driven by priorities or institutions in the Global North that collect empirical data in the Global South (Kolinjivadi et al., 2023).

### Research designs, methods, and evidence

The case studies we included in our analyses were peer‐reviewed journal articles (*n* = 125; 69%), reviews (*n* = 44; 24%), reports (*n* = 8; 4%), book chapters (*n* = 4; 2%), or working papers (*n* = 1; 0.5%). Of the 125 case study articles (e.g., studies that used qualitative or quantitative approaches to draw inference about a particular site), 72 used a cross‐sectional research design (58%) and 20 used a longitudinal design (16%). Counterfactual studies (*n* = 1; 0.8%) and studies drawing on model predictions (*n* = 1; 0.8%) represented the smallest proportion of articles, as did descriptive case studies (*n* = 12; 9.6%). The remaining 19 articles (15.2%) drew on a combination of approaches.

The 125 case studies captured in the systematic map spanned ecological and social disciplines and many were inter‐ or multidisciplinary. Almost half of studies (*n* = 52; 41%) employed social research methods, such as focus groups, interviews, and reviews, whereas those with an ecological focus employed mostly forest inventories and plots, satellite data, and soil sampling (*n* = 17; 13.6%). Interdisciplinary studies that drew on mixed ecological field methods and social science methods, such as IFRI methods (International Forestry Resources and Institutions methods), were most commonly represented (*n* = 56; 44.8%). Studies providing mixed qualitative and quantitative evidence of ecological outcomes of conservation on lands held by IP&LC were most commonly represented (*n* = 57; 45.6%), followed by studies drawing on qualitative (*n* = 28; 22.4%) and studies drawing on quantitative evidence (*n* = 14; 11.2%). One study included subjective self‐reporting, where the authors were self‐assessing the success of their own work but did not provide independent evidence to support their conclusions. Conceptual frameworks were explicitly used or developed in 7 of the case studies to represent the expected or detected relationship between drivers and ecological outcomes.

### Enabling conditions

The enabling conditions identified were divided into 2 broad categories: first, national and international factors beyond the control of local communities (e.g., contextual factors) (*n* = 131) or that relate to the implementation of conservation interventions by external actors (*n* = 84), and, second, factors related to community characteristics that may affect how effectively they can conserve biodiversity (*n* = 164) (Table 2). Of the 182 papers, 153 discussed the 20 enabling conditions identified as supporting effective conservation on IP&LC land.

**TABLE 2:** Number of articles out of 182 that reported on different conditions that enable positive conservation outcomes on Indigenous and community lands.

Factors external to local communities	Description	Number of papers
Background enabling conditions		
Law and policy	Government rules, regulations, and policy measures, i.e.,	90
	legal land status (e.g., land rights and tenure type),	67
	natural resource‐use regulations,	24
	protected designations	21
Governance	Conditions related to governance by external actors, i.e., accountability, bureaucracy, corruption, enforcement, political will, respect for traditional practices and trust between stakeholders	65
Environmental	Conditions related to the biophysical properties of a site, e.g., forest type, elevation, accessibility	51
Economic forces	Conditions related to the selling and purchasing of goods and services, including access to markets and certification and the availability of technology	47
Land and resource use	Transformations of the natural landscape outside of lands held by Indigenous Peoples and local communities, including through agriculture and resource extraction by noncommunity members	40
Demography	Conditions related to the structure of human populations, i.e., population density, population growth, migration, distance from roads	36
Enabling conditions related to external conservation interventions		
Funding	Financial inputs (i.e., incentives or compensation) received by Indigenous Peoples and local communities from external sources	60
Empowerment	Measures designed to increase the degree of autonomy and self‐determination in Indigenous Peoples and local communities	52
Capacity building	Actors affect Indigenous Peoples and local communities by increasing their capacity to practice conservation	23
Benefit sharing	When an intervention is designed to compensate Indigenous Peoples and local communities for products or services generated on their lands	15
Duration of commitment	Period of time during which external actors maintain their involvement with Indigenous Peoples and local communities	12

**Factors internal to Indigenous People and local communities**		
Community governance	Ways in which Indigenous Peoples and local communities manage themselves through social norms, i.e.,	96
	enforcement	49
	organization	39
	ownership and stewardship	39
	common pool resource governance	34
	traditional decision‐making structures	23
Forms of land and resource use	Ways in which Indigenous Peoples and local communities extract value from their land and forest resources, i.e.,	83
	resource extraction	65
	forest management	56
	agricultural practices	50
Socioeconomic characteristics	Community size, income level, degree of educational attainment, ethnicity, infrastructure, household characteristics, and practice of tourism within lands held by Indigenous Peoples and local communities.	60
Connection to nature	Conditions that describe how Indigenous Peoples and local communities perceive and value nature, i.e., species preferences, spiritual connection and sense of identity	51
Dependency on forest resources	Degree to which the subsistence of Indigenous Peoples and local communities are tied to natural resources and the use of technologies, such as efficient cookstoves, gas or electricity, that diminish their use of traditional fuels	41
Community law and policy	Customary land status	35
Traditional and local knowledge	Knowledge held by Indigenous Peoples and local communities about their land and its resources, and how to manage them	32
Legal and media capacity	Capacity, including Indigenous Peoples and local communities’ awareness of and ability to interact with their broader legal and mediatic context	17
Historical experience	Having lived and acknowledged the effects of conditions such as environmental degradation	17

The most frequently occurring contextual factors concerned the broad political context at the national and international scale, including law and policy (*n* = 90) (e.g., government rules, regulations, and policy measures) and governance (*n* = 65) (e.g., issues relating to accountability, bureaucracy, corruption, enforcement, and political will). Strong community governance also emerged as the most frequently discussed community factor (*n* = 96), followed by community land and resource use (*n* = 83). The presence of governance and law and policy as both contextual and community factors reflected the different scales at which these key factors operate. For example, community law and policy refers to customary land rights, whereas community governance refers to the ways in which IP&LC manage themselves through social norms.

Several key subfactors emerged under community governance, with the most frequently discussed being the ability of communities to enforce their own rules and regulations, particularly around natural resource use and the illegal extraction of resources on their lands by those outside the community. Although deemed an important condition for ecological outcomes of conservation in many of the papers included in this study, the presence of traditional decision‐making structures was the least discussed subfactors under community governance.

Land and resource use was the second most commonly discussed community factor, particularly the resource extraction patterns of local communities. In contrast, fewer than half the papers explored the role of land and resource use by external parties, including the illegal extraction of resources by noncommunity members.

Overall, 5 factors relating to externally devised conservation interventions were identified. However, these factors were the least discussed overall, particularly the role of benefit sharing, capacity building, and the duration of commitment needed for conservation interventions to be successful.

### Ecological outcomes of conservation

Ecological outcomes of conservation were organized into 2 top‐level categories: those related to ecosystems and those related to species (Table 3). Of the 182 studies, 153 discussed 11 different ecological outcomes of conservation on IP&LC‐held forested lands. The majority of articles measured or described ecosystem outcomes (*n* = 143), primarily in terms of forest cover, followed by forest quality and water quality or availability. Outcomes such as soil quality were not given as much attention as other ecological outcomes of conservation. Carbon sequestration was the least well‐studied ecological outcome.

**TABLE 3:** Number of articles that discuss (*n* = 182) ecosystem‐related outcomes and species outcomes in our review of the different conditions that enable positive conservation outcomes on Indigenous and community lands.

Ecosystems	Description	No. of papers
Forest cover	The land area that is covered by forest or forest canopy	118
Forest quality	The health and productivity of the forest	71
Water quality or availability	The condition of water as it relates to its contextual purpose (sustaining life) and its abundance and accessibility	39
Soil quality	The capacity of soil function to sustain biological productivity	24
Erosion rates	Action of wind, water and other natural agents that remove soil	24
Carbon sequestration	The process by which carbon dioxide is removed from the atmosphere and held in solid or liquid form	16

Species		
Diversity	Number of species and their distribution within the forest ecosystem	51
Population trends	Changes to distribution, size of population and life histories of species within the forest	37
Richness	Number of species found in a forest ecosystem	19
Threatened	Effects on the populations of species listed as threatened on the International Union for Conservation of Nature Red List	15
Invasive	Non‐native species present in the forest	11

*Note*: Articles can appear in more than one category.

Species outcomes were discussed in 74 of the articles, with over half the number of studies examining ecosystem‐level outcomes. Papers predominantly explored species diversity and species population trends in relation to either community or contextual drivers of change. The impact of drivers of change on threatened species or on the presence of invasive species was the least commonly examined species‐level outcome. Of the 153 articles examining ecological outcomes of conservation, 64 studies looked at both ecosystem‐level and species‐level outcomes.

The distribution and relative frequency of linkages between enabling conditions and ecological outcomes of conservation across the literature included in this study are presented in Table 4. Of the 182 papers, 147 explored both the enabling conditions and ecological outcomes of conservation on IP&LC‐held lands. Ecosystem‐level outcomes were more frequently linked to enabling conditions (*n* = 137). Particularly, the role of governance (both as a contextual and community factor) in forest cover and forest quality was frequently emphasized. Community land and resource use was the second factor most frequently linked with ecological outcomes of conservation, namely with forest cover (*n* = 64).

**TABLE 4:** Number of articles in which a link was made between different ecological outcomes of conservation and reported factors that enable positive conservation outcomes on Indigenous and community lands.

	Ecological outcome												
	Ecosystems	Forest cover	Forest quality	Water quality or availability	Erosion rates	Soil quality	Carbon sequestration	Species	Species diversity	Population trends	Species richness	Invasive species	Threatened species
**Factors external to local communities**													
Background enabling conditions													
Law and policy		59	18	8	9	3	5		16	5	5	1	1
Governance		37	39	2	3		2		4	1	1		
Land and resource use		36	14	3	3	1	3		3	3	2	1	1
Demographic		30	9	2	3	1	2		5	2	3	2	2
Economic forces		16	7		1	1	1		3	4	2	1	
Environmental		14	5		2				4	2	1		
Enabling conditions related to externally devised conservation interventions													
Empowerment		26	13	4	3	2	2		8	3	3	2	4
Funding		24	18	1	4	2	2		2	4	1	2	1
Capacity building		6	3	4		3				1			1
Benefit sharing		5	2		3		1		1		1		
Duration of commitment		2	2						1	2			1
**Factors internal to local communities**													
Community governance		67	25	4	8	2	2		10	4	4	1	2
Land and resource use		63	39	13	14	12	6		33	18	14	6	1
Socioeconomic characteristics		29	13	3	6	2	4		6	4	1	1	1
Connection to nature		27	17	4	4	4	2		15	6	6	1	
Dependency on forest resources		25	12	1	1	1	1		3	3		1	
Community law and policy		17	3		2		1		2				
Traditional and local knowledge		15	5	1		2			9		1		1
Legal and media capacity		9	8	1	1								
Historical experience		5	6	1	1		1		2	1			

*Note*: Articles can appear in more than one category.

Although customary land status was commonly discussed (Table 2), there were fewer studies that explicitly linked the role of customary land status with ecological outcomes of conservation. Law and policy was the most commonly linked contextual factor. Customary status was the sixth most commonly linked community factor, ranking after socioeconomic characteristics, connection to nature, and dependency of the community on forest cover and quality.

Other linkages were less studied. For example, community factors were more focused on issues of local governance and resource use, whereas the roles of traditional or local ecological knowledge and historic experience were less frequently studied. Ecosystem‐level outcomes such as water and soil quality and carbon sequestration were infrequently linked to enabling conditions, relative to forest cover and quality.

Species‐level outcomes were less commonly linked to enabling conditions compared with the ecosystem‐level outcomes (*n* = 63). In particular, studies linking enabling conditions to the presence or absence of invasive species or the conservation of threatened species were lacking. Species‐level outcomes were more frequently linked with community factors compared to contextual factors. Particularly, the role of community governance, land and resource use, and connection to nature were more commonly identified. Community law and policy was also less commonly associated with species‐level outcomes than it is with ecosystem‐level outcomes.

## DISCUSSION

We documented which conditions are identified in the sampled literature as contributing to different environmental outcomes on forested lands held by IP&LC. In a time of increasing global environmental change, there is a need to better support Indigenous and local governance structures to address social and ecological challenges. We have added to the growing literature that seeks to evidence how IP&LC can best be supported in their conservation efforts. We built on previous studies, such as Dawson et al. (2021), who examined the conditions for conservation outcomes more broadly but did not identify specific ecological outcomes of conservation, as we tried to do here. Our resultant systematic map highlights several gaps and biases in the literature, including a high representation of a few countries, and linkages between a small number of enabling conditions and ecological outcomes of conservation. Although we aimed to elucidate gaps and biases in the kinds of evidence being collected to support the important role of IP&LC‐led conservation efforts for ecological outcomes, we recognize the challenges of aligning such conservation initiatives with purely biophysical science criteria and hope that Indigenous approaches to conservation can be recognized independently (see Gilchrist et al., 2005; Brook & McLachlan, 2005).

The volume of articles published increased from the year 2002, a trend shown in other systematic maps and reviews of the role of nature in human well‐being and the role of IP&LC in equitable conservation (Busch & Ferretti‐Gallon, 2023; Dawson et al., 2021; McKinnon et al., 2016). This may be because international investment in forest conservation and management increased in the 1990s, which directed funding to sustainable forest management and reduction of emissions from deforestation (Agrawal et al., 2013; Miller, 2013). It may also reflect a partial shift away from fortress conservation and toward community‐based approaches and consideration of human well‐being (Brittain et al., 2021), supported by a substantial shift in global environmental policy toward more inclusive approaches (Newing & Perram, 2019).

The enabling conditions we identified fell in 2 main categories: contextual conditions, which are beyond the control of communities and the absence of which often limits the ability of communities to conserve (e.g., legal land title), and conditions related to the characteristics and practices of the communities themselves that affect whether and how effectively they act if the necessary contextual conditions are in place (e.g., intact local decision‐making structures, maintenance of Indigenous knowledge). These categories align loosely with 2 dominant discourses in conservation: external intervention and behavior change. These discourses often emphasize community characteristics and actions as a threat to conservation (Brandon et al., 1998; Brockington, 2002) and collaboration and partnership, which emphasizes the potential for communities to conserve, given appropriate contextual enabling conditions (Brechin et al., 2003; Brosius et al., 2005).

We identified some cross‐cutting categories, such as governance and law and policy, that were important contextually and within communities. Secure legal land status for communities was the contextual condition most commonly associated with positive conservation outcomes. At the community level, a community's ability to enforce its own rules, self‐organize, and have a sense of ownership and stewardship was the condition most commonly associated with positive environmental outcomes. These findings support those of Dawson et al. (2021) that conservation outcomes are improved where IP&LC play a central role, such as when they have substantial influence over decision‐making or when local institutions regulating tenure form a recognized part of governance. This also aligns with Ostrom's (1990) design principles on collective action and resource management and the finding of Persha et al. (2011) that local participation in rulemaking enhances conservation and livelihood outcomes in forested areas. The importance of local decision‐making is a particularly important point to reiterate in this study because there exist long‐standing viewpoints in conservation that emphasize the role of market‐based approaches and income generation over people‐centered conservation (Sandbrook et al., 2019).

We also found some stark contrasts between how frequently the role of community and national or international conditions were addressed. For example, more than twice as many included papers identified community land and resource use as a factor, compared to studies exploring the role of wider land‐ and resource‐use change (e.g., transformations of the natural landscape outside IP&LC lands, including through resource extraction by noncommunity members). The studies that examined community behaviors set out to document and demonstrate the environmental stewardship or impact of communities regarding their agricultural activities, forest management, and nontimber forest product use. Coupled with the focus on the ability of IP&LC to enforce local laws under community governance, this points to a bias in the literature that emphasizes preventing environmental degradation on IP&LC‐held lands firmly on the communities themselves, with little emphasis on preventing degradation by land‐use change and illegal resource extraction outside of community control. However, over 25% of IP&LC lands could face increasingly high pressure in the future from commodity‐driven development (WWF et al., 2021) and from so‐called green environmental agendas (Fairhead et al., 2012). More research on the wider socioecological system that recognizes and evidences the impact of contextual land‐use change on biodiversity within IL&LC‐held lands would be a welcome focus.

The potential role of community characteristics such as land and resource use and governance was also more commonly addressed than local knowledge or historic experiences. As the post‐2020 Global Biodiversity Framework recommends the application of both Western science and traditional knowledge and practices (CBD, 2022), more focus on the role of community characteristics, including knowledge, beliefs, and traditional decision‐making structures that can contribute to conservation, would be particularly relevant.

Our division of governance, law and policy, and land‐ and resource‐use conditions that relate to both community and contextual factors could open up a more balanced discussion about rights and responsibilities at different scales (i.e., human rights need to be recognized, but this recognition of human rights needs to be complemented by strong community governance and cohesion). This division is supported in previous work. For example, Robinson et al. (2017) point out in their response to Blackman et al. (2017) that local land titling does not always lead to full land rights and as such both the enabling national legislative context and the community context are required to ensure communities have secure rights to their land. Further, our division of community and national or international governance conditions support the idea that although community cohesion and organization are key conditions related to governance, they may be insufficient without the timely and competent support of national authorities to enable communities to effectively exercise their rights (Wilkie & Painter, 2021).

The majority of studies measured or described ecosystem outcomes, primarily in terms of forest cover; considerably fewer assessed species outcomes. A focus on measuring forest cover may be expected, given our focus on forest habitats. Although recent advances in open‐access products have helped establish remote sensing as a vital tool for understanding how land cover and land use are changing over time (Hill et al., 2019), forest cover is a poor surrogate for biodiversity value (Tropek et al., 2014). This is especially pertinent as evidence continues to point to the increases in empty forest syndrome in tropical forests (Bogoni et al., 2022). Although articles in our study have contributed evidence to the role of IP&LC in conserving mammals (O'Bryan et al., 2020; Zimmerman et al., 2018), more studies that use an integrated approach to combine forest cover and biodiversity metrics are needed to more holistically inform policy and local decision‐making (Hill et al., 2019) and to assess the ecological impacts of social forestry (Bong et al., 2019; Brockerhoff et al., 2017). Numerous studies highlight the conditions under which community management tends to be successful, but they almost all focus on local livelihood benefits and do not explicitly address globally valued goods, such as biodiversity (Sayer et al., 2017). The growing emphasis on conservation on IP&LC‐lands means that participatory biodiversity monitoring will become increasingly important and will work best if it is designed jointly based on community as well as external values and priorities (Chandler et al., 2017; Jager et al., 2020; Shirk et al., 2012) and if great care is taken to apply ethical codes of conduct (Sandbrook et al., 2018; Sharma et al., 2020). Our systematic map also showed how ecological outcomes of conservation such as soil and water quality, which disproportionately affect IP&LC (Shelton, 2013), are not being given as much attention as other ecological outcomes in the academic literature.

Our systematic map showed an overall lack of studies that set out to provide causal evidence between the enabling conditions discussed and the conservation outcomes measured, reflecting other similar findings from systematic studies (Calder et al., 2020; Cheng et al., 2019). More specific follow‐up reviews and carefully designed empirical studies are needed to provide evidence of the impacts of the different potential enabling conditions we identified. This in turn can improve understanding of what actions and support will help deliver specific conservation outcomes in future, and can contribute evidence that can help ensure existing human rights laws are adhered to. In particular, robust studies that provide evidence for both changes in ecological indicators on IP&LC lands, aside from forest cover, and the factors that contribute to these outcomes are needed to close the gap between policy and practice and direct effective funding and support.

There is a lack of studies looking at the effectiveness of different community‐based conservation approaches. These will be sorely needed as recognition grows for the need to move away from protected areas alone and support conservation on lands owned and managed by IP&LC. The target of protecting 30% of Earth's surface by 2030 (30×30) by establishing a combination of protected areas and other effective conservation measures (OECMs), which include IP&LC‐held lands outside of protected areas, was recently written into the Kunming–Montreal post‐2020 Global Biodiversity Framework. However, the target does not currently state what proportion of this 30% must come from OECMs and does not guarantee that the rights of IP&LC will be respected and promoted in the process (Worsdell et al., 2020). Woodhouse et al. (2022) examined the assumptions behind 5 key narratives about the role of protected areas in conservation and found evidence for the narratives that participation in conservation and secure tenure rights support effective conservation, with limited support for the narratives that protected areas are propoor and reducing poverty supports conservation. More research is needed to improve understanding of what changes and conditions best support IP&LC, especially given the focus on rights‐based approaches in the post‐2020 Global Biodiversity Framework.

The collaborative work of 3 different researchers permitted the review of a large number of titles, abstracts, and full texts within a limited timeframe. Discrepancies in the coding decisions of each member of the research team may have resulted in some minor inconsistencies in coding, but we are confident that joint deliberations by the research team on any cases where the coding was not straightforward minimized these.

Additionally, conservation, unlike other fields such as medicine, lacks standard ontology, and the terms that may be used when IP&LC contribute to or lead conservation may differ from those used in mainstream conservation. Although we attempted to ensure that our search strategy captured this diversity by validating our search terms through contacts with expertise in environmental policy and human rights, failing to include specific terms in our search may have resulted in some areas of literature being missed. Additionally, the search was limited to English language only, given restraints in terms of time and resources. A great deal of literature exists in other languages, and further research could seek to replicate the review in other languages, which would improve the quality and validity of results by widening the evidence base geographically (Konno et al., 2020) and recognizing and amplifying the knowledge being produced in local languages (North et al., 2020).

Our systematic map showed the quantity and distribution of articles captured in our data set that measure ecological outcomes of conservation on forested lands held by IP&LC and identified the enabling conditions discussed as important. We did not assess the underlying quality of individual articles because we did not conduct a critical appraisal. As such, the higher occurrences of links between certain enabling conditions and certain conservation outcomes do not imply a higher quality of evidence. Rather, it demonstrates only that this link is well addressed in the literature included in this study.

In our view, the concepts related to enabling conditions and the types of evidence available are not currently standardized enough to readily lend themselves to a quantitative meta‐analysis. However, a useful follow‐on would be to do this analysis for individual enabling conditions and their ecological and social outcomes, based on more focused literature searches. Such studies can also offer critical lessons on the potential pitfalls and challenges of conservation implementation by considering disabling as well as enabling conditions. Since we conducted our review, Dawson, Coolsaet, Bhardwai, Booker et al. (2024) published an excellent review of this kind that examines the links between one enabling condition—community autonomy or control—and ecological and social outcomes.

Future research could also prioritize the integration of socioeconomic outcomes of IP&LC‐led conservation alongside ecological outcomes. Understanding how enabling conditions affect not only the ecology but also the social, economic, and cultural well‐being of IP&LC, including not only governance (which is well addressed in Dawson, Coolsaet, Bhardwaj, Booker, et al. [2024]) but also factors such as land rights, livelihoods, cultural rejuvenation, and intergenerational knowledge transfer, could further highlight the role of these factors in more successful conservation efforts. These connections could also be explored empirically through longitudinal studies to assess the long‐term and contextual impacts of enabling conditions and through comparative analyses across different geographical regions and cultural contexts to uncover region‐specific challenges and opportunities. These steps could inform the development of new, context‐sensitive strategies and policy adaptations that ultimately support more effective and equitable IP&LC‐led conservation initiatives.

Our systematic map highlighted critical gaps in understanding the impact of IP&LC on forest conservation and the factors that have been reported in the literature as potentially contributing to enabling their effective stewardship. In particular, our map identified a prevalent focus on certain community characteristics, such as their use of land and resources as drivers of conservation outcomes; a lack of consideration of the role of other internal factors, such as traditional knowledge, beliefs, and decision‐making structures; and a lack of emphasis on external socioecological forces and responsibilities at national and international scales. There is also a need for evidence on changes in ecological indicators other than forest cover, including soil and water quality, carbon sequestration, biodiversity metrics, and the factors that contribute to these outcomes in IP&LC‐held lands. Future research should address these knowledge gaps and expand analyses of ecological outcomes to include socioeconomic variables and insights from other disciplines. Ideally, the type and quality of the evidence supporting each of these links need to be critically appraised. This would enable policymakers, practitioners, and IP&LC themselves to better target their conservation actions.

The broad range of enabling conditions for conservation on IP&LC lands we identified may reflect how different scholars choose to focus on different enabling conditions and conservation outcomes based on their research fields and preconceptions (Fariss et al., 2013). Future research could help to develop a framework of both community and contextual factors to examine multiple conditions and their impacts on the effectiveness of conservation actions, for example, based on qualitative, field‐based studies.

The unique knowledge and practices of IP&LC are invaluable to sustainable conservation, offering insights crucial for contemporary and future strategies. Recognizing and integrating their rights into environmental policies is urgent for achieving global environmental goals and targets, including the 23 targets of the Kunming–Montreal Global Biodiversity Framework, UN Sustainable Development Goals, and the Paris Agreement. Our map provides an overview of key factors with robust links to positive ecological outcomes, which can be translated into policy priorities for supporting more effective and equitable IP&LC‐led conservation efforts across a wide range of contexts. It also serves as a benchmark for identifying future directions for this growing body of research, which can ultimately contribute to the development of context‐sensitive strategies and policies to support Indigenous‐led conservation initiatives and inform funding distribution to support IP&LC in their vital role in global conservation efforts.

## AUTHOR CONTRIBUTIONS

*Conceptualization*: Helen Newing and Stephanie Brittain. *Data collection and analysis*: Leigh‐Anne Bullough, Andrea Alatorre, and Stephanie Brittain. *Writing*: Stephanie Brittain and Helen Newing. *Comments and edits on drafts*: Helen Newing, Leigh‐Anne Bullough, Andrea Alatorre, and Stephanie Brittain.

## Supporting information



AppendixS1‐S3

Supporting Information

## References

[cobi70055-bib-0001] Agrawal, A. (2001). Common property institutions and sustainable governance of resources. World Development, 29(10), 1649–1672.

[cobi70055-bib-0002] Agrawal, A. , Cashore, B. , Hardin, R. , Sheperd, G. , Benson, C. , & Miller, D. (2013). Economic contributions of forests (Background Paper 1). United Nations Forum on Forests.

[cobi70055-bib-0003] Amano, T. , Berdejo‐Espinola, V. , Christie, A. P. , Willott, K. , Akasaka, M. , Báldi, A. , Berthinussen, A. , Bertolino, S. , Bladon, A. J. , Chen, M. , Choi, C. Y. , Bou Dagher Kharrat, M. , de Oliveira, L. G. , Farhat, P. , Golivets, M. , Hidalgo Aranzamendi, N. , Jantke, K. , Kajzer‐Bonk, J. , Kemahlı Aytekin, M. Ç. , … Sutherland, W. J. (2021). Tapping into non‐English‐language science for the conservation of global biodiversity. PLoS Biology, 19(10), Article e3001296. 10.1371/journal.pbio.3001296 34618803 PMC8496809

[cobi70055-bib-0004] Blackman, A. , Corral, L. , Lima, E. S. , & Asner, G. P. (2017). Titling indigenous communities protects forests in the Peruvian Amazon. Proceedings of the National Academy of Sciences of the United States of America, 114(16), 4123–4128. 28373565 10.1073/pnas.1603290114 PMC5402456

[cobi70055-bib-0005] Bogoni, J. A. , Ferraz, K. M. P. M. B. , & Peres, P. (2022). Continental‐scale local extinctions in mammal assemblages are synergistically induced by habitat loss and hunting pressure. Biological Conservation, 272, Article 109635.

[cobi70055-bib-0006] Bong, I. W. , Moeliono, M. , Wong, G. Y. , & Brockhaus, M. (2019). What is success? Gaps and trade‐offs in assessing the performance of traditional social forestry systems in Indonesia. Forest and Society, 3(1), 1–21.

[cobi70055-bib-0007] Brandon, K. , Redford, K. H. , & Sanderson, S. (Eds.). (1998). Parks in peril: People, politics, and protected areas. Island Press.

[cobi70055-bib-0008] Brechin, S. , Wilshusen, P. , Fortwrangler, C. , & West, P. (Eds.). (2003). Contested nature: Promoting international biodiversity conservation with social justice in the twenty‐first century. State University of New York Press.

[cobi70055-bib-0009] Brittain, S. , Tugendhat, H. , Newing, H. , & Milner‐Gulland, E. (2021). Conservation and the rights of Indigenous peoples and local communities: Looking forwards. Oryx, 55(5), 641–642.

[cobi70055-bib-0010] Brockerhoff, E. G. , Barbaro, L. , Castagneyrol, B. , Forrester, D. I. , Gardiner, B. , González‐Olabarria, J. R. , Lyver, P. O'. B. , Meurisse, N. , Oxbrough, A. , Taki, H. , Thompson, I. D. , van der Plas, F. , & Jactel, H. (2017). Forest biodiversity, ecosystem functioning and the provision of ecosystem services. Biodiversity Conservation, 26, 3005–3035.

[cobi70055-bib-0011] Brockington, D. (2002). Fortress conservation: The preservation of the Mkomazi Game Reserve. Indiana University Press.

[cobi70055-bib-0012] Brooks, J. , Waylen, A. , & Borgerhoff Mulder, M. (2012). How national context, project design, and local community characteristics influence success in community‐based conservation projects. Proceedings of the National Academy of Sciences of the United States of America, 109(52), 21265–21270. 23236173 10.1073/pnas.1207141110 PMC3535631

[cobi70055-bib-0012a] Brook, R. K. , & McLachlan, S. M. (2005). On using expert‐based science to “Test” local ecological knowledge. Ecology and Society, 10(2). http://www.jstor.org/stable/26267765

[cobi70055-bib-0013] Brosius, P. , Tsing, A. , & Zerner, C. H. (Eds.). (2005). Communities and conservation: Histories and politics of community‐based natural resource management. Altamira.

[cobi70055-bib-0013a] Bryant, A. , & Charmaz, K. (2007). The SAGE handbook of grounded theory. SAGE Publications Ltd.

[cobi70055-bib-0014] Busch, J. , & Ferretti‐Gallon, K. (2023). What drives and stops deforestation, reforestation, and forest degradation? An updated meta‐analysis. Review of Environmental Economics and Policy, 17(2), 217–250.

[cobi70055-bib-0015] Calder, R. S. D. , Alatorre, A. , Marx, R. S. , Mallampalli, V. , Mason, S. A. , Olander, L. P. , Jeuland, M. , & Borsuk, M. E. (2020). Graphical models and the challenge of evidence‐based practice in development and sustainability. Environmental Modelling & Software, 130, Article 104734.

[cobi70055-bib-0016] Chandler, M. , See, L. , Copas, K. , Bonde, A. M. Z. , López, B. C. , Danielsen, F. , Legind, J. K. , Masinde, S. , Miller‐Rushing, A. J. , Newman, G. , Rosemartin, A. , & Turak, E. (2017). Contribution of citizen science towards international biodiversity monitoring. Biological Conservation, 213, 280–294.

[cobi70055-bib-0016a] Charmaz, K. (2006). Constructing Grounded Theory: A Practical Guide through Qualitative Analysis. London: Sage Publications.

[cobi70055-bib-0017] Cheng, S. H. , LacLeod, K. , Ahlroth, S. , Onder, S. , Perge, E. , Shyamsundar, P. , Rana, P. , Garside, R. , Kristjanson, P. , McKinnon, M. C. , & Miller, D. C. (2019). A systematic map of evidence on the contribution of forests to poverty alleviation. Environmental Evidence, 8, Article 3.

[cobi70055-bib-0018] Coase, R. H. (1960). The problem of social cost. The Journal of Law & Economics, 56, 837–877.

[cobi70055-bib-0019] Convention of Biological Diversity (CBD) . (2022). Post‐2020 Global Biodiversity Framework, draft recommendation submitted by the Co‐Chairs . Author.

[cobi70055-bib-0020] Corson, C. , Worcester, J. , Rogers, S. , & Flores‐Ganley, I. (2020). From paper to practice? Assembling a rights‐based conservation approach. Journal of Political Ecology, 27(1), 1128–1147.

[cobi70055-bib-0021] Dawson, N. , Coolsaet, B. , Bhardwaj, A. , Booker, F. , Brown, D. , Lliso, B. , Loos, J. , Martin, A. , Oliva, M. , Pascual, U. , Sherpa, P. , & Worsdell, T. (2024). Is it just conservation? A typology of Indigenous peoples’ and local communities’ roles in conserving biodiversity. One Earth, 7(6), 1007–1021. 10.1016/j.oneear.2024.05.001

[cobi70055-bib-0022] Dawson, N. , Coolsaet, B. , Bhardwaj, A. , Brown, D. , Lliso, B. , Loos, J. , Mannocci, L. , Martin, A. , Oliva, M. , Pascual, U. , Sherpa, P. , & Worsdell, T. (2024). Reviewing the science on 50 years of conservation: Knowledge production biases and lessons for practice. Ambio, 53, 1395–1413. 39023682 10.1007/s13280-024-02049-w PMC11383897

[cobi70055-bib-0023] Dawson, N. , Coolsaet, B. , Sterling, E. , Loveridge, R. , Gross‐Camp, N. D. , Wongbusarakum, S. , Sangha, K. K. , Scherl, L. M. , Phuong Phan, H. , Zafra‐Calvo, N. , Lavey, W. G. , Byakagaba, P. , Julián Idrobo, C. , Chenet, A. , Bennett, N. J. , Mansourian, S. , & Rosado‐May, F. J. (2021). The role of Indigenous peoples and local communities in effective and equitable conservation. Ecology and Society, 26(3), Article 19.

[cobi70055-bib-0024] Fa, J. E. , Watson, J. E. M. , Leiper, I. , Potapov, P. , Evans, T. D. , Burgess, N. D. , Molnár, Z. , Fernández‐Llamazares, Á. , Duncan, T. , Wang, S. , Austin, B. J. , Jonas, H. , Robinson, C. J. , Malmer, P. , Zander, K. K. , Jackson, M. V. , Ellis, E. , Brondizio, E. S. , & Garnett, S. T. (2020). Importance of Indigenous Peoples’ lands for the conservation of Intact Forest Landscapes. Frontiers in Ecology and the Environment, 18(3), 135–140.

[cobi70055-bib-0025] Fairhead, J. , Leach, M. , & Scoones, I. (2012). Green grabbing: A new appropriation of nature? Journal of Peasant Studies, 39(2), 237–261.

[cobi70055-bib-0024a] Fariss, B. , DeMello, N. , Powlen, K. A. , Latimer, C. E. , Masuda, Y. , & Kennedy, C. M. (2023). Catalyzing success in community‐based conservation. Conservation Biology. 10.1111/cobi.13973 PMC10087706 35796041

[cobi70055-bib-0026] Garnett, S. T. , Burgess, N. D. , Fa, J. E. , Fernández‐Llamazares, Á. , Molnár, Z. , Robinson, C. J. , Watson, J. E. M. , Zander, K. K. , Austin, B. , Brondizio, E. S. , Collier, N. F. , Duncan, T. , Ellis, E. , Geyle, H. , Jackson, M. V. , Jonas, H. , Malmer, P. , McGowan, B. , Sivongxay, A. , & Leiper, I. (2018). A spatial overview of the global importance of Indigenous lands for conservation. Nature Sustainability, 1, 369–374.

[cobi70055-bib-0025a] Gilchrist, G. , Mallory, M. , & Merkel, F. (2005). Can local ecological knowledge contribute to wildlife management? Case studies of migratory birds. Ecology and Society, 10(1), 20. http://www.ecologyandsociety.org/vol10/iss1/art20/

[cobi70055-bib-0026a] Glaser, B. , & Strauss, A. (1967). The Discovery of Grounded Theory: Strategies for Qualitative Research. Mill Valley, CA: Sociology Press.

[cobi70055-bib-0027] Haddaway, N. R. , Bernes, C. , Jonsson, B. G. , & Hedlund, K. (2016). The benefits of systematic mapping to evidence‐based environmental management. Ambio, 45, 613–620. 26984257 10.1007/s13280-016-0773-x PMC4980318

[cobi70055-bib-0028] Hatcher, J. , Owen, M. , & Yin, D. (2021). Falling short: Donor funding for indigenous peoples and local communities to secure tenure rights and manage forests in tropical countries (2011‐2020) . Rainforest Foundation Norway.

[cobi70055-bib-0029] Hill, R. , Grant, C. , George, M. , Robinson, C. J. , Jackson, S. , & Abel, N. (2012). A typology of indigenous engagement in Australian environmental management: Implications for knowledge integration and social‐ecological system sustainability. Ecology and Society, 17(1), Article 23.

[cobi70055-bib-0030] Hill, S. L. L. , Arnell, A. , Maney, C. , Butchart Stuart, H. M. , Hilton‐Taylor, C. , Ciciarelli, C. , Davis, C. , Dinerstein, E. , Purvis, A. , & Burgess, N. D (2019). Measuring forest biodiversity status and changes globally. Frontiers in Forests and Global Change, 2, Article 70.

[cobi70055-bib-0031] Huber‐Stearns, H. R. , Bennett, D. E. , Posner, S. , Richards, R. C. , Fair, J. H. , Cousins, S. J. M. , & Romulo, C. L. (2017). Social‐ecological enabling conditions for payments for ecosystem services. Ecology and Society, 22(1), Article 18.

[cobi70055-bib-0032] Intergovernmental Science‐Policy Platform on Biodiversity and Ecosystem Services (IPBES) . (2019). Global assessment report on biodiversity and ecosystem services of the Intergovernmental Science‐Policy Platform on Biodiversity and Ecosystem Services. IPBES Secretariat.

[cobi70055-bib-0033] Jack, B. K. , Kousky, C. , & Sims, K. (2008). Designing payments for ecosystem services: Lessons from previous experience with incentive‐based mechanisms. Proceedings of the National Academy of Sciences of the United States of America, 150(28), 9465–9470. 10.1073/pnas.0705503104 PMC2474507 18621696

[cobi70055-bib-0034] Jager, N. W. , Newig, J. , Challies, E. , & Kochskämper, E. (2020). Pathways to implementation: Evidence on how participation in environmental governance impacts on environmental outcomes. Journal of Public Administration Research and Theory, 30(3), 383–399.

[cobi70055-bib-0035] James, K. L. , Randall, N. R. , & Haddaway, N. R. (2016). A methodology for systematic mapping in environmental sciences. Environmental Evidence, 5, Article 7.

[cobi70055-bib-0036] Kolinjivadi, V. , Van Hecken, G. , & Merlet, P. (2023). A largely Northern‐driven agenda: A systematic review of 15 years of knowledge generation on payments for ecosystem services (PES). *SSRN Electronic Journal*. 10.2139/ssrn.4340568

[cobi70055-bib-0037] Konno, K. , Akasaka, M. , Koshida, C. , Katayama, N. , Osada, N. , Spake, R. , & Amano, T. (2020). Ignoring non‐English‐language studies may bias ecological meta‐analyses. Ecology and Evolution, 10(13), 6373–6384. 32724519 10.1002/ece3.6368 PMC7381574

[cobi70055-bib-0038] McKinnon, M. C. , Cheng, S. H. , Dupre, S. , Edmond, J. , Garside, R. , Glew, L. , Holland, M. B. , Levine, E. , Masuda, Y. J. , Miller, D. C. , Oliveira, I. C. , Revenaz, J. , Roe, D. , Shamer, S. , Wilkie, D. , Wongbusarakum, S. , & Woodhouse, E. (2016). What are the effects of nature conservation on human well‐being? A systematic map of empirical evidence from developing countries. Environmental Evidence, 5, Article 8.

[cobi70055-bib-0039] Miller, D. C. (2013). Explaining global patterns of international aid for linked biodiversity conservation and development. World Development, 59, 341–359.

[cobi70055-bib-0040] Miller , D. C. , Mansourian, S. , & Wildburger, C. (2020). Forests, trees and the eradication of poverty: Potential and limitations. A Global Assessment Report (IUFRO World Series Volume 39). International Union of Forest Research Organizations (IUFRO).

[cobi70055-bib-0041] Munn, Z. , Peters, M. D. J. , Stern, C. , Tufanaru, C. , McArthur, A. , & Aromataris, E. (2018). Systematic review or scoping review? Guidance for authors when choosing between a systematic or scoping review approach. BMC Medical Research Methodology, 18, Article 143. 30453902 10.1186/s12874-018-0611-x PMC6245623

[cobi70055-bib-0042] Newing, H. , & Perram, A. (2019). What do you know about conservation and human rights? Oryx, 53(4), 595–596.

[cobi70055-bib-0043] Newton, P. , Kinzer, A. T. , Miller, D. C. , Oldekop, J. A. , & Agrawal, A. (2020). The number and spatial distribution of forest‐proximate people globally. One Earth, 3(3), 363–370.

[cobi70055-bib-0044] Norström, A. V. , Cvitanovic, C. , & Löf, M. F. (2020). Principles for knowledge co‐production in sustainability research. Nature Sustainability, 3, 182–190.

[cobi70055-bib-0045] North, M. A. , Hastie, W. W. , & Hoyer, L. (2020). Out of Africa: The underrepresentation of African authors in high‐impact geoscience literature. Earth‐Science Reviews, 208, Article 103262.

[cobi70055-bib-0046] O'Bryan, C. J. O. , Garnett, S. T. , Fa, J. E. , Leiper, I. , Rehbein, J. A. , Fernández‐Llamazares, A. , Jackson, M. V. , Jonas, H. D. , Brondizio, E. S. , Burgess, N. D. , Robinson, C. J. , Zander, K. K. , Molnár, Z. , Venter, O. , & Watson, J. E. M. (2020). The importance of Indigenous Peoples’ lands for the conservation of terrestrial mammals. Conservation Biology, 35(3), 1002–1008. 32852067 10.1111/cobi.13620 PMC8247428

[cobi70055-bib-0047] Ostrom, E. (1990). Governing the commons: The evolution of institutions for collective action. Cambridge University Press.

[cobi70055-bib-0048] Ostrom, E. (2009). A general framework for analyzing sustainability of social‐ecological systems. Science, 325, 419–422. 19628857 10.1126/science.1172133

[cobi70055-bib-0049] Persha, L. , Agrawal, A. , & Chhatre, A. (2011). Social and ecological synergy: Local rulemaking, forest livelihoods, and biodiversity conservation. Science, 331, 1606–1608. 21436453 10.1126/science.1199343

[cobi70055-bib-0050] Pullin, A. S. , Frampton, G. K. , Livoreil, B. , & Petrokofsky, G. (2022). Guidelines and standards for evidence synthesis in environmental management. Version 5.1 . Collaboration for Environmental Evidence.

[cobi70055-bib-0051] QSR International Pty Ltd . (2018). NVivo qualitative data analysis software (Version 12) . https://www.qsrinternational.com/nvivo‐qualitative‐data‐analysis‐software/home

[cobi70055-bib-0052] Rands, M. R. W. , Adams, W. M. , Bennun, L. , Butchart, S. H. M. , Clements, A. , Coomes, D. , Entwistle, A. , Hodge, I. , Kapos, V. , Scharlemann, J. P. , Sutherland, W. J. , & Vira, B. (2010). Biodiversity conservation: Challenges beyond 2010. Science, 329, 1298–1303. 20829476 10.1126/science.1189138

[cobi70055-bib-0053] Raymond, C. M. , Bryan, B. A. , MacDonald, D. H. , Cast, A. , Strathearn, S. , Grandgirard, A. , & Kalivas, T. (2009). Mapping community values for natural capital and ecosystem services. Ecological Economics, 68, 1301–1315.

[cobi70055-bib-0054] Robinson, B. E. , Holland, M. B. , & Naughton‐Treves, L. (2017). Community land titles alone will not protect forests. Proceedings of the National Academy of Sciences of the United States of America, 114(29), Article E5764. 28645894 10.1073/pnas.1707787114 PMC5530705

[cobi70055-bib-0055] Sabatier, P. A. (1986). Top‐down and bottom‐up approaches to implementation research: A critical analysis and suggested synthesis. Journal of Public Policy, 6(1), 21–48.

[cobi70055-bib-0056] Sandbrook, C. , Fisher, J. A. , Holmes, G. , Luque‐Lora, R. , & Keane, A. (2019). The global conservation movement is diverse but not divided. Nature Sustainability, 2, 316–323.

[cobi70055-bib-0057] Sandbrook, C. , Luque‐Lora, R. , & Adams, W. (2018). Human bycatch: Conservation surveillance and the social implications of camera traps. Conservation and Society, 16(4), 493–504.

[cobi70055-bib-0058] Sayer, J. , Margules, C. , & Boedhihartono, A. K. (2017). Will biodiversity be conserved in locally‐managed forests? Land, 6(1), Article 6.

[cobi70055-bib-0059] Sharma, K. , Fiechter, M. , George, T. , Young, J. , Alexander, J. S. , Bijoor, A. , Suryawanshi, K. , & Mishra, C. (2020). Conservation and people: Towards an ethical code of conduct for the use of camera traps in wildlife research. Ecological Solutions and Evidence, 1(2), Article e12033. 10.1002/2688-8319.12033

[cobi70055-bib-0060] Shelton, D. (2013). Water rights of indigenous peoples and local communities. In L. B. de Chazournes , C. Leb , & M. Tignino (Eds.), International law and freshwater: The multiple challenges (pp. 69–94). Edward Elgar Publishing.

[cobi70055-bib-0061] Shirk, J. L. , Ballard, H. L. , Wilderman, C. C. , Phillips, T. , Wiggins, A. , Jordan, R. , McCallie, E. , Minarchek, M. , Lewenstein, B. V. , Krasny, M. E. , & Bonney, R. (2012). Public participation in scientific research: A framework for deliberate design. Ecology and Society, 17(2), Article 29. 10.5751/ES-04705-170229

[cobi70055-bib-0062] Smith, T. , Bulkan, J. , Zerriffi, H. , & Tansey, J. (2019). Indigenous peoples, local communities, and Payments for Ecosystem Services. The Canadian Geographer /Le Géographe Canadien, 63(4), 616–630.

[cobi70055-bib-0063] Snilstveit, B. , Oliver, S. , & Vojtkova, M. (2012). Narrative approaches to systematic review and synthesis of evidence for international development policy and practice. Journal of Development Effectiveness, 4(3), 409–429.

[cobi70055-bib-0064] Strauss, A. , & Corbin, J. (1994). Grounded theory methodology: An overview. In N. Denzin & Y. Lincoln (Eds.), Handbook of qualitative research (pp. 273–284). Sage.

[cobi70055-bib-0065] Tauli‐Corpuz, V. , Alcorn, J. , Molnar, A. , Healy, C. , & Barrow, E. (2020). Cornered by PAs: Adopting rights‐based approaches to enable cost‐effective conservation and climate action. World Development, 130, Article 104923.

[cobi70055-bib-0066] Tengö, M. , Brondizio, E. S. , Elmqvist, T. , Malmer, P. , & Spierenburg, M. (2014). Connecting diverse knowledge systems for enhanced ecosystem governance: The multiple evidence base approach. Ambio, 43(5), 579–591. 24659474 10.1007/s13280-014-0501-3 PMC4132468

[cobi70055-bib-0067] Timko, J. , Le Billon, P. , Zerriffi, H. , Honey‐Rosés, J. , de la Roche, I. , Gaston, C. , Sunderland, T. C. H. , & Kozak, R. A. (2018). A policy nexus approach to forests and the SDGs: Tradeoffs and synergies. Current Opinion in Environmental Sustainability, 34, 7–12.

[cobi70055-bib-0068] Tropek, R. , Sedlácek, O. , Beck, J. , Keil, P. , Musilová, Z. , Símová, I. , & Storch, D. (2014). Comment on “High‐resolution global maps of 21st‐century forest cover change”. Science, 344, 981. 10.1126/science.1248753 24876487

[cobi70055-bib-0069] Walcott, J. , Harris, M. , Beard, S. , Labbate, G. , Miles, L. , & Kapos, V. (2020). Making good on the Glasgow Climate Pact: A call to action to achieve one gigaton of emissions reductions from forests by 2025. United Nations Environment Programme (UNEP‐WCMC).

[cobi70055-bib-0070] Wilkie, D. , & Painter, M. (2021). Factors of success in community forest conservation. Conservation Science and Practice, 3(5), Article e388. 10.1111/csp2.388

[cobi70055-bib-0071] Woodhouse, E. , Bedelian, C. , Barnes, P. , Cruz‐Garcia, G. S. , Dawson, N. , Gross‐Camp, N. , Homewood, K. , Jones, J. P. G. , Martin, A. , Morgera, E. , & Schreckenberg, K. (2022). Rethinking entrenched narratives about protected areas and human wellbeing in the Global South. UCL Open Environment, 4, Article e050. 37228477 10.14324/111.444/ucloe.000050 PMC10208335

[cobi70055-bib-0072] World Wide Fund for Nature (WWF), UN Environment Programme World Conservation Monitoring Centre (UNEP‐WCMC), GEF Small Grants Programme, ICCA‐Global Support Initiative (SGP/ICCA‐GSI), LandMark Global Platform of Indigenous and Community Lands (LM), The Nature Conservancy (TNC), Conservation International (CI), Wildlife Conservation Society (WCS), UNDP Equator Prize (EP), International Land Coalition Secretariat (ILC‐S), Conservation Matters LLC (CM), & International Union for Conservation of Nature (IUCN) . (2021). The State of Indigenous Peoples’ and Local Communities’ Lands and Territories: A technical review of the state of Indigenous Peoples’ and Local Communities’ lands, their contributions to global biodiversity conservation and ecosystem services, the pressures they face, and recommendations for actions . https://wwflac.awsassets.panda.org/downloads/report_the_state_of_the_indigenous_peoples_and_local_communities_lands_and_territories_1.pdf

[cobi70055-bib-0073] Worsdell, T. , Kumar, K. , Allan, J. R. , Gibbon, G. E. M. , White, A. , Khare, A. , & Frechette, A. (2020). Rights based conservation: The path to preserving Earth's biological and cultural biodiversity? Rights & Resources. https://hdl.handle.net/11245.1/3e4229e7‐6e2b‐459d‐a720‐6287108ccf7f

