# Supplementary material for: Enabling conditions for conservation on Indigenous and community lands
Source: Conserv Biol. 2025 Jun 19;40(1):e70055. doi: 10.1111/cobi.70055 (PMC12856806; doi:10.1111/cobi.70055)
Supplement: Supplementary file 1 — AppendixS1‐S3 [file COBI-40-e70055-s001.docx]

**Appendix, S1:** Codebook produced during the systematic map of the enabling conditions for conservation outcomes on Indigenous or community-held forested lands.

| **Enabling conditions** | **Conditions that affect the capacity of IP&LC to practise conservation in their lands** |
| --- | --- |
| **Contextual factors** | **Factors that are outside of the control of IP&LC** |
| ***Background enabling conditions*** | |
| **Demography** | Conditions related to the structure of human populations, i.e. population density, population growth, migration, distance from roads. |
| **Economic forces** | Conditions related to the supply and demand of goods and services. |
| Access to markets | The ability of IP&LC to sell or purchase goods and services. |
| Certification | Externally awarded recognition of good practices for the production of goods and services. |
| Market forces | Pressures exerted on land-use decisions by the price, supply and demand of goods and services. |
| Available technology | The existence and availability of technological advances. |
| **Environmental** | Conditions related to the biophysical properties of a site, i.e. forest type, elevation, accessibility, forest condition, size of site. |
| **Governance** | Conditions related to governance by external actors. |
| Accountability | Acknowledgement and assumption of responsibilities from external actors. |
| Bureaucracy | Administrative procedures that support or hinder positive ecological outcomes. |
| Corruption | When powerful external actors engage in dishonest or fraudulent conduct. |
| Enforcement | External actors’ ability to ensure compliance with rules or regulations. |
| Political will | Commitment from government agencies or officials. |
| Respect for traditional practices | External recognition of the rights of IP&LC to maintain and use their traditional practices |
| Trust between stakeholders | The ability of stakeholders to work collaboratively and maintain relationships with IP&LC |
| **Land and resource use** | Transformations of the natural landscape and resource extraction outside of IP&LC control. |
| Conversion to farmland | Conversation of land to farmland. |
| Resource extraction by non-community members | When resources – i.e. wildlife, timber, NTFPs, minerals or land itself – that are found within IP&LC lands are extracted by non-members |
| **Law & Policy** | Government sanctioned rules, regulations and courses or action |
| Legal land status | The conditions under which land is owned and managed |
| *Access rights* | *Legally recognized rights to enter forested lands* |
| *Exclusion rights* | *Legally recognized rights to prevent non-members from accessing forested lands* |
| *Management rights* | *Legally recognized rights to regulate the use of forests and their resources* |
| *Ownership rights* | *Certainty that IP&LC rights to land will be recognized and protected* |
| *Property rights* | *Legal recognition of the rights of IP&LC to their lands, including access, exclusion, management, ownership and use rights* |
| *Use rights* | *Legally recognized rights to extract forest resources* |
| Protected designations | When a territory is legally recognized as dedicated to conservation |
| Natural resource use regulations | Rules and regulations designed to control the use of forested lands and their resources |
| ***Enabling conditions related to external conservation interventions*** | |
| **Benefit sharing** | When an intervention is designed to compensate IP&LCs for products or services generated on their lands |
| **Capacity building** | Actors affect IP&LCs by increasing their capacity to practise conservation, including the availability of labour and employment generation from conservation |
| **Duration of commitment** | The period of time during which external actors maintain their involvement with IP&LCs |
| **Empowerment** | Measures designed to increase the degree of autonomy and self-determination in IP&LCs, including economic, educational, political, psychological and social empowerment |
| **Funding** | Financial inputs – i.e. availability of funding and incentives or compensation – received by IP&LCs from external sources. |
| **Factors internal to IP&LC** | **Actions or circumstances caused by factors within IP&LC control or influence** |
| **Community law & policy** | Community sanctioned rules, regulations and courses or action, such as customary land status and legitimacy of rules and laws. |
| **Legal and media capacity** | IP&LCs awareness of and ability to interact with their broader legal and mediatic context |
| **Connection to nature** | Conditions that describe how IP&LC perceive and value their natural resources, i.e. species preferences, spiritual connection and sense of identity |
| **Dependency on forest resources** | The degree to which the subsistence of IP&LC is tied to natural resources and the use of technologies, such as efficient cookstoves, gas or electricity, that diminish their use of traditional fuels |
| **Community governance** | The ways in which IP&LC manage themselves through social norms. |
| Enforcement | IP&LC ability to ensure compliance with internal rules |
| Organisation | The ability of IP&LC to organise to fulfil common goals |
| Ownership and stewardship | The perception that forests or their resources are owned and cared for by IP&LC |
| Common-pool resource institutions | The local institutions that IP&LCs use to manage their communal resources, i.e. assemblies, regulations, democracy, community service |
| Traditional decision-making structures | The institutions through which IP&LCs make decisions about common issues |
| **Historic experience** | Having lived and acknowledged the effects of conditions such as environmental degradation |
| **Socio-economic characteristics** | Community size, income level, degree of educational attainment, ethnicity, infrastructure, household characteristics and the presence of tourism or ecotourism within IP&LC lands. |
| **Traditional and local knowledge** | Knowledge held by IP&LCs about their land and its resources, and how to manage them |
| **Forms of land and resource use** | The ways in which IP&LC make use of their land |
| Agricultural practices | Methods employed by IP&LCs for growing crops and raising animals |
| Forest management practices | The methods employed by IP&LCs for making use of forests and their resources |
| Resource extraction by community members | When resources that are found within IP&LC lands are extracted by community members |
| **Outcomes** | **Consequences or changes that result from enabling conditions** |
| **Ecological outcomes** | **Consequence of actions for an ecosystem and its component parts** |
| ***Ecosystems*** | |
| **Carbon sequestration** | The process by which carbon dioxide is removed from the atmosphere and held in solid or liquid form |
| **Forest cover** | The land area that is covered by forest or forest canopy |
| **Forest quality** | The health and value of the forest |
| **Soil quality** | The capacity of soil function to sustain biological productivity |
| **Erosion rates** | Action of wind, water and other natural agents that remove soil |
| **Water quality or availability** | The condition of the water as it relates to its contextual purpose (sustaining life) and the abundance and accessibility of the water |
| ***Species*** | |
| **Invasive species** | Non-native species present in the forest |
| **Population trends** | Changes to distribution, size of population and life histories of species within the forest |
| **Threatened species** | Effects on the populations of species listed as threatened on the IUCN Red List |
| **Species diversity** | The number of species and their distribution within the forest ecosystem |
| **Species richness** | Number of species found in a forest ecosystem |
| **Evidence** | **How outcomes were measured or determined** |
| **Methods** | **The procedure for accomplishing the collection of evidence** |
| ***Ecological field methods*** | |
| **Biological inventories** | An ongoing effort to catalogue the flora and fauna in an area in its current state and as the ecosystem changes over time |
| **Forest inventory plots** | Systematic collection of ecological data from determined areas of a forest to calculate the ecological standard for the forest as a whole |
| **Ground truthing** | A method of checking the accuracy of data by means of in-situ observations |
| **Quadrats** | Using a number of small areas, selected at random, to act as samples for assessing qualities of a wider area |
| **Satellite data** | The use of information about earth gathered by man-made satellites in orbit |
| **Soil sampling** | The principle of collecting a portion of soil to determine the status of said soil e.g. nutrient status |
| **Transects** | A path along which one counts and records occurrences of the species of study |
| **Vegetation surveys** | A method used to define vegetation types and assess plant species present in the population |
| ***Interdisciplinary*** | |
| **IFRI** | A particular method of collecting ecological, socio-economic and institutional data about a forest system |
| **Mixed methods** | Studies that draw upon both ecological and social methods |
| ***Social Research Methods*** | |
| **Focus groups** | Collective information from a group of people that fit pre-defined criteria to discuss a particular topic |
| **Interviews** | Qualitative research technique which involves conducting intensive individual discussions with respondents who meet predefined criteria |
| **Rapid inventories** | Scientists and IP&LC collaborate to identify patterns of social organisation and opportunities for capacity building |
| **Review of reports or documents** | The analysis of information gathered from reviewing reports or documents |
| **Social surveys** | The collection of information from a sample of individuals through their responses to questions |
| **Systematic review** | A detailed and comprehensive plan and search strategy used to gather information from a body of relevant literature |
| **Direct observation** | Collecting information by watching the subject in their usual environment, without the researcher altering said environment |
| **Study design** | **The set of methods and procedures used to collect and analyse data** |
| **Counterfactuals** | Contrasting sites or conditions were studied in order to determine what would have happened in the absence of an intervention |
| **Cross-sectional** | Two or more variables were measured in order to statistically determine a relationship between them |
| **Longitudinal** | A type of correlational research that looks at variables over an extended period of time |
| **Model predictions** | Mathematical methods rather than measurements were used to obtain results |
| **Descriptive** | Single or comparative case studies are used to draw inferences. |
| **Mixed survey design** | A combination of approaches is employed |
| **Type of evidence** | **A broad categorization of the kind of data used to support statements** |
| **Qualitative** | Evidence was produced through inquiry of local knowledge |
| **Quantitative** | Evidence was produced through measurements or numerical estimates |
| **Mixed** | Evidence is produced from both qualitative and quantitative sources |
| **NA** | The evidence presented is deemed unreliable by the coders based on its source or methods |

**Appendix, S2:** Journals from which at least three articles were identified in the systematic map of the enabling conditions for conservation outcomes on Indigenous or community-held forested lands.

| **Journal name** | **Number of articles** |
| --- | --- |
| Ecology and Society | 10 |
| Environmental Conservation | 9 |
| Biodiversity and Conservation | 6 |
| Conservation Biology | 6 |
| Journal of Environmental Management | 6 |
| Environmental Management | 5 |
| Forests | 5 |
| Biological Conservation | 4 |
| Conservation Science and Practice | 4 |
| Forest Ecology and Management | 4 |
| Forest Policy and Economics | 4 |
| International Forestry Review | 4 |
| Journal of Sustainable Forestry | 4 |
| Land | 4 |
| Land Use Policy | 4 |
| Society & Natural Resources | 4 |
| Biodiversity | 3 |
| Global Environmental Change | 3 |
| PNAS | 3 |

**Appendix, S3:** Reference list for all articles included in this systematic map and the ecological and enabling conditions coded to each article (See attached .csv spreadsheet).
